# Machine Learning-Based Routine Laboratory Tests Predict One-Year Cognitive and Functional Decline in a Population Aged 75+ Years

**DOI:** 10.3390/brainsci13040690

**Published:** 2023-04-20

**Authors:** Karina Braga Gomes, Ramon Gonçalves Pereira, Alexandre Alberto Braga, Henrique Cerqueira Guimarães, Elisa de Paula França Resende, Antônio Lúcio Teixeira, Maira Tonidandel Barbosa, Wagner Meira Junior, Maria das Graças Carvalho, Paulo Caramelli

**Affiliations:** 1 Faculdade de Farmácia, Universidade Federal de Minas Gerais, Belo Horizonte 31270-901, MG, Brazil; 2 Instituto de Ciências Exatas, Universidade Federal de Minas Gerais, Belo Horizonte 31270-901, MG, Brazil; 3 Faculdade de Medicina, Universidade Federal de Minas Gerais, Belo Horizonte 31270-901, MG, Brazilcaramelli@ufmg.br (P.C.); 4 Hospital das Clínicas (EBSERH), Universidade Federal de Minas Gerais, Belo Horizonte 31270-901, MG, Brazil; 5 Faculdade Santa Casa BH, Belo Horizonte 30110-005, MG, Brazil

**Keywords:** machine learning, cognitive decline, functional decline, laboratory variables

## Abstract

Background: Cognitive and functional decline are common problems in older adults, especially in those 75+ years old. Currently, there is no specific plasma biomarker able to predict this decline in healthy old-age people. Machine learning (ML) is a subarea of artificial intelligence (AI), which can be used to predict outcomes Aim: This study aimed to evaluate routine laboratory variables able to predict cognitive and functional impairment, using ML algorithms, in a cohort aged 75+ years, in a one-year follow-up study. Method: One hundred and thirty-two older adults aged 75+ years were selected through a community-health public program or from long-term-care institutions. Their functional and cognitive performances were evaluated at baseline and one year later using a functional activities questionnaire, Mini-Mental State Examination, and the Brief Cognitive Screening Battery. Routine laboratory tests were performed at baseline. ML algorithms—random forest, support vector machine (SVM), and XGBoost—were applied in order to describe the best model able to predict cognitive and functional decline using routine tests as features. Results: The random forest model showed better accuracy than other algorithms and included triglycerides, glucose, hematocrit, red cell distribution width (RDW), albumin, hemoglobin, globulin, high-density lipoprotein cholesterol (HDL-c), thyroid-stimulating hormone (TSH), creatinine, lymphocyte, erythrocyte, platelet/leucocyte (PLR), and neutrophil/leucocyte (NLR) ratios, and alanine transaminase (ALT), leukocyte, low-density lipoprotein cholesterol (LDL-c), cortisol, gamma-glutamyl transferase (GGT), and eosinophil as features to predict cognitive decline (accuracy = 0.79). For functional decline, the most important features were platelet, PLR and NLR, hemoglobin, globulin, cortisol, RDW, glucose, basophil, B12 vitamin, creatinine, GGT, ALT, aspartate transferase (AST), eosinophil, hematocrit, erythrocyte, triglycerides, HDL-c, and monocyte (accuracy = 0.92). Conclusions: Routine laboratory variables could be applied to predict cognitive and functional decline in oldest-old populations using ML algorithms.

## 1. Introduction

Population aging is a worldwide phenomenon [1]. An aging process free of any cognitive, behavioral, and motor impairment allows for better integration of older adults in society. However, aging is associated with the occurrence of disabilities, partly due to chronic-degenerative conditions such as dementia [2,3].

Dementia is a term that encompasses a particular group of symptoms, including difficulties with memory, language, problem solving, and other skills that affect an individual’s ability to perform routine activities [4]. Nevertheless, a significant amount of the older adult population is affected by age-related cognitive and functional decline, regardless of dementia [5]. 

Machine learning (ML) is a subarea of artificial intelligence (AI) applied in many technological fields, including healthcare. ML uses training inputs and outputs to generate a function that can be used to predict outcomes in other inputs. The application of ML in the prediction of dementia may provide an advantage to health care by promoting a better understanding of the pathophysiology of the disease and a more accurate diagnosis [6,7]. 

Currently, there is no specific plasma biomarker able to predict cognitive or functional decline in healthy old-age people and cerebrospinal fluid markers are only measured when dementia is suspected. Conversely, routine laboratory tests are accessible for these populations and are applied habitually to the elderly. The hypothesis is that routine laboratory tests could predict impairment in a population that is most likely to present cognitive and functional decline (75+ years) using ML models, which could detect complex patterns and interactions between these biomarkers. As a result, the ML algorithm selected a total of 20 laboratory variables that are able to predict cognitive and functional decline in the oldest population. The data suggest that alterations in laboratory variables may be associated with an individual’s cognitive and functional status and ML models could find the best combination of these routine blood exams, which is not possible in a univariate analysis. As far as we know, there has been no study investigating routine laboratory markers as predictors of cognitive and functional decline in an older adult population.

## 2. Material and Methods

### 2.1. Characteristics of the Population 

The participants were selected from the Pietà study, a population-based research project conducted in Caeté City, Southeast Brazil [3]. Individuals aged 75+ years, which are most likely to have decreased cognitive and functional capacity due to age, were actively searched through community-health public-program agents or in long-term-care institutions, as well as announcements on local radio and newspapers [8]. 

Functional assessments of the subjects were carried out by the functional activities questionnaire (FAQ) [9], while cognitive performance was evaluated through brief cognitive tests, including the Mini-Mental State Examination and the Brief Cognitive Screening Battery [10]. Functional and cognitive tests were applied twice (2008 and 2009), with a one-year interval, and normalized by schooling level (Z score). The Z scores derived from each cognitive test were used to calculate a global cognitive score (GCS) [11]. The baseline characteristics of this population were described elsewhere [3]. No participant presented cognitive or functional impairment at the baseline.

According to the mathematical difference between GCS or FAQ scores obtained in 2008 and 2009, the subjects were then categorized into two groups: decliners, who showed a short-term cognitive or functional decline after one-year follow up (negative score); and nondecliners, those who demonstrated short-term stability or even improvement (positive score) in cognitive or functional abilities after one year.

The study was approved by the Ethics Committee of the Federal University of Minas Gerais, Brazil and all participants or their legally acceptable representatives signed the written informed consent.

### 2.2. Biochemical Analysis 

Blood samples were collected from the participants, after fasting for 12 h, for routine laboratory tests at the baseline. The variables that composed the dataset were: complete blood count (CBC), triglycerides, red blood cell distribution width (RDW), high-density lipoprotein cholesterol (HDL-c), low-density lipoprotein cholesterol (LDL-c), neutrophil-lymphocyte ratio (NLR), platelet-lymphocyte ratio (PLR), fasting glucose, aspartate transaminase (AST), alanine transaminase (ALT), creatinine, albumin, globulin, cortisol, B12 vitamin, thyroid-stimulating hormone (TSH) and gamma-glutamyl transferase (GGT).

The blood count was performed on ABX Pentra Horiba^®^ (Madrid, Spain). The lipid profile was analyzed with Super Class—Labtest^®^ (Lagoa Santa, Brazil) equipment. The other parameters were determined using DPC Bayer (Whippany, NJ, USA) or VITROS OrthoClinical Diagnostics^®^ (Singapure City, Singapure), following the manufacturer’s recommendations. 

### 2.3. Data Analysis

For the characterization of the population and individual analysis of the variables, the normality was verified by the Shapiro–Wilk test. Differences between the groups were evaluated using Student’s *t*-Test. Statistical analyses were conducted using SPSS software version 21 (New York City, NY, USA). Any *p* values < 0.05 were considered significant.

In order to predict cognitive and functional decline, three supervised-learning algorithms were applied: random forest, XGBoost, and support vector machine (SVM). Random forest and XGBoost are a combination of models that aim to generate prediction models through a set of data interconnected by decision trees. The combination of the average results of these links determines the class of results that better matches the outcome. Random forest trains each tree independently, based on a random sampling of the data, while XGBoost builds the trees sequentially, in which each new tree corrects the errors of the previous one, improving the accuracy of the final results. SVM uses binary classification methods that list the variables based on their probability to present a high grade of significance for the model. SVM is based on the geometric properties of the data; through a kernel function, the data are mapped to a multidimensional space to seek a hyperplane that better divides the classes [12].

**Figure 1:**
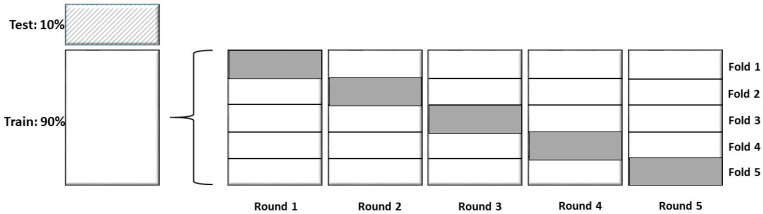
Diagram showing the dataset analysis. First, models from the random combination of 90% of all variables were performed. Then, for the models with better accuracy, cross validation was applied: the data from all patients were divided by 5; 4 folds were used for training and the last fold was used for testing—this process was repeated five times. The final model was tested with 10% of the data. All the metrics are reported as the average of these tests. Gray rectangle: test folder.

For the application of the models, a Scikit-Learn was performed, using Python software version 3.6.9 (Wilmington, DE, USA). We used the following steps:

Step 1: Data preparation. The preprocessing step included standardization of the units of measurement and class balancing. In order to reduce the bias caused by unbalanced classes, the SMOTE oversampling technique was used for training data, in which the synthetic samples of the minority class were created using the k-nearest neighbors of an instance (k = 3). The number of results generated was the value enough to match both groups;

Step 2: Model training. All of the features (laboratory tests) were used for iterative training. In this step, features were replaced continuously in order to optimize the model hyperparameters and reduce prediction errors. To establish the best classification algorithm, 90% of the data set was selected randomly as training and the remaining 10% was used for the testing set. Different values of the number of estimators and depth of the tree for the random forest and XGBboost algorithms and different cost values and kernel functions for SVM were tested (Supplementary Material—Table S1) in order to maximize the area under the ROC curve (AUROC) and the accuracy and to avoid sampling bias or data overfitting. The AUROC was calculated by varying the decision threshold and then measuring the false-positive and true-positive rates for each of them [13,14];

Step 3. Internal model validation. The models were trained with cross-validation k fold (k = 5). The data set was subdivided into k folds, and, at each time, k-1 folds were used for training and the rest for validation (Figure 1). 

In addition, in order to estimate the performance of each classifier, the accuracy, sensitivity, and specificity were evaluated. A feature selection was implemented to identify the most relevant subset of variables for predicting cognitive and functional decline [13], based on the number of times that a variable was assessed by the algorithm decision. The information about the variables used by the model was assessed by the Shapley additive explanations (SHAP) [15], an effective model interpretation that helps to better understand the relationship between variables. The effect of each variable was calculated by the distance from the original prediction. A mean of absolute distances for each individual was used to generate a ranked list of variables according to the effect on outcome predictions for all subjects. A negative distance was obtained if the individual had a value that reduces the risk of cognitive/functional impairment compared to the average risk of the population studied, while a positive distance was obtained when the risk in favor of cognitive/functional decline was higher than the population evaluated. An average of absolute distances between variables was used to generate a ranking list according to the effect on the decline prediction.

## 3. Results

### 3.1. Model Performance

Of the 132 participants, who were predominantly low-educated rural individuals, the mean age was 79.52 ± 4.26 years old and 51 were male. Cognitive and functional declines were observed in 53% and 36% of the participants, respectively, after the one-year follow up. Age and gender frequency were not different between the groups (*p* > 0.05) (Table 1). 

The univariate comparisons of the variables between the groups are shown in the Supplementary Material—Table S2. Individuals with functional decline presented lower leukocyte values and PLR, but higher lymphocyte values and ALT levels compared to nondecliners (*p* < 0.05). For cognitive impairment, no difference was observed between the groups (all *p* > 0.05). Only 16% of the individuals presented both cognitive and functional declines; therefore, it was not possible to evaluate them separately. 

Comparing the three models, random forest and XGBoost showed the best performance to predict cognitive decline (accuracy = 0.79 for both) when compared to the SVM model (accuracy = 0.71). For functional decline, random forest presented higher accuracy (0.92) when compared to the XGBoost and SVM models (0.83 and 0.71, respectively), in addition to higher sensitivity and specificity values (Supplementary Material—Table S3). Accordingly, random forest presented better prediction performance in both groups, in addition to allowing for the setting of the variables of major importance for the models. 

### 3.2. One-Year Follow-Up Cognitive Decline Predicted by the Random Forest Model

**Figure 2:**
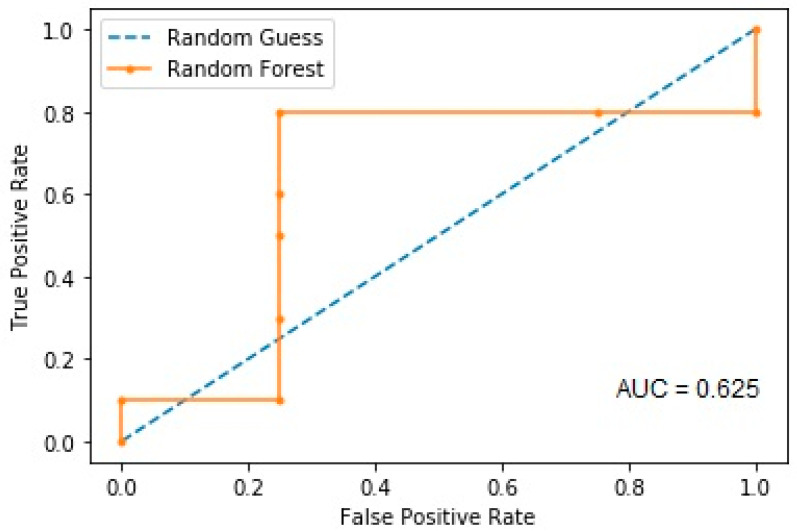
Area under the ROC curve for the prediction of cognitive decline.

The random forest model presented an AUROC of 0.625 for the prediction of cognitive decline (Figure 2).

The importance of the feature of each variable within the model was estimated using the SHAP algorithm. For each individual, the effect of the absence of the variable was calculated using the distance from the original prediction. A negative distance was obtained if the individual had a value that reduced the risk of cognitive impairment compared to the average risk of the population studied and a positive distance was obtained when the risk in favor of cognitive decline was higher than the population evaluated. An average of absolute distances between variables was used to generate a ranking list according to the effect on the decline prediction.

**Figure 3:**
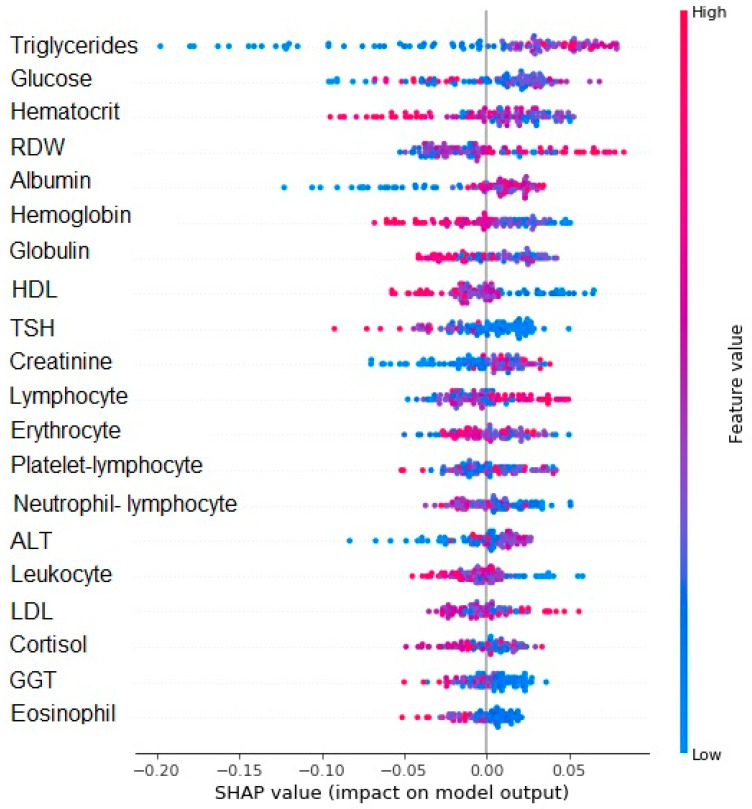
SHAP value for the random forest model regarding cognitive decline. Values below 0 on the X axis tend to not decline and values above 0 tend to show cognitive decline. The red color indicates the greater value of the variable, and the blue color indicates the lower value.

The SHAP algorithm showed that in decreasing order of importance, triglycerides, glucose, hematocrit, RDW, albumin, hemoglobin, globulin, HDL, TSH, creatinine, lymphocyte, erythrocyte, PLR and NLR, ALT, leukocyte, LDL, cortisol, GGT, and eosinophil were the variables considered most important for the model. Values below zero on the X axis tended to contribute to cognitive stability, while values above zero tended to contribute to cognitive decline after one year. The red color indicated higher values of the variable and the blue color indicated lower values of the variable. Considering the 10 variables with greater feature values (Y axis), higher triglycerides, RDW, albumin, and creatinine levels, as well as lower glucose, hematocrit, hemoglobin, globulin, HDL, and TSH levels are associated with cognitive decline (Figure 3).

### 3.3. One-Year Follow-Up Functional Decline Predicted by the Random Forest Model

**Figure 4:**
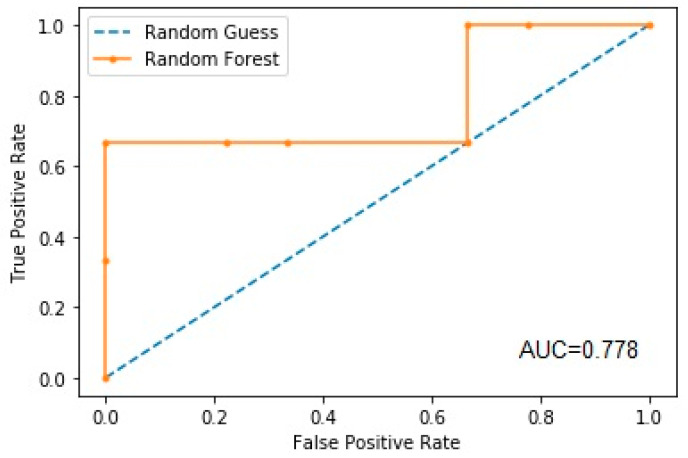
Area under the ROC curve for the prediction of functional decline.

The AUROC for the prediction of functional decline by the random forest model was 0.778 (Figure 4).

**Figure 5:**
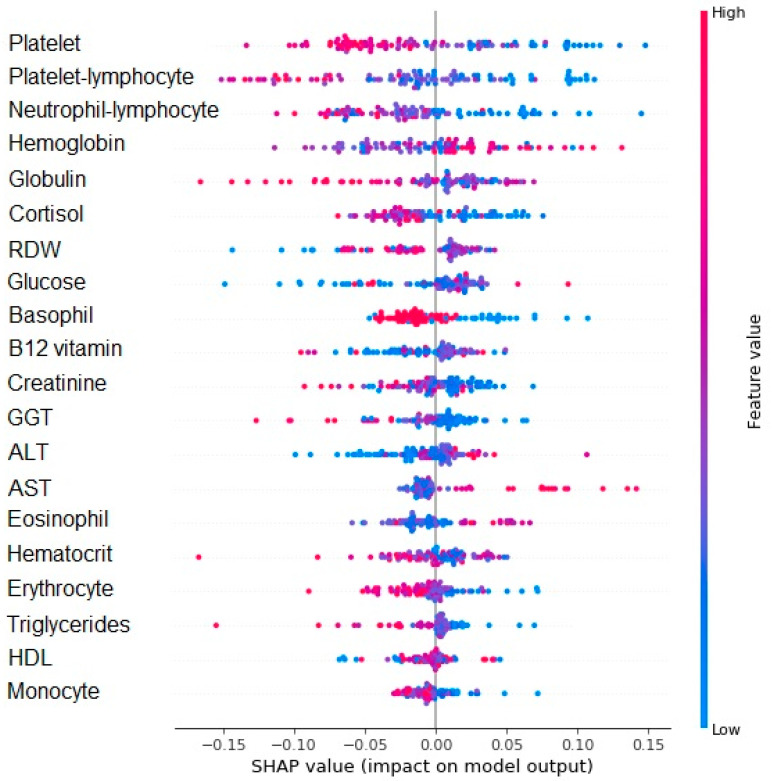
SHAP value for the random forest model regarding functional decline. Values below 0 on the X axis tend to not decline and values above 0 tend to cognitive decline. The red color indicates the greater value of the variable and the blue color indicates the lower value.

The feature importance of each variable within the model was also estimated using the SHAP algorithm. The variables in decreasing order of importance were platelet, PLR and NLR, hemoglobin, globulin, cortisol, RDW, glucose, basophil, B12 vitamin, creatinine, GGT, ALT, AST, eosinophil, hematocrit, erythrocyte, triglycerides, HDL, and monocyte. Similar to the previous interpretation, values below zero on the X axis tended to contribute to the nonfunctional decline. Values above zero were associated with functional decline in a one-year follow up. The red color indicates higher values and the blue color indicates lower values for the variable. The 10 variables with higher feature values (Y axis) related to functional decline were higher hemoglobin levels and lower platelet-lymphocyte and neutrophil-lymphocyte ratios, platelet, globulin, cortisol, RDW, glucose, basophil, and vitamin B12 levels (Figure 5).

## 4. Discussion

The final model selected a total of 20 variables that are able to predict cognitive and functional decline in a nondementia oldest-old population, with an accuracy of 0.79 and 0.92, respectively. Currently, the results of state-of-the-art studies with mild cognitive impairment (MCI) patients undergoing neuroimaging tests showed that the best-performing method reached an accuracy from 0.42 to 0.63 [16,17].

In the present study, regarding red-blood-cell parameters, lower values were observed for hematocrit and hemoglobin and higher values for RDW, among the 10 most important variables for cognitive decline, which sets up a tendency to anemia. Erythrocyte counts also showed lower values in those with cognitive decline but did not appear among the 10 most important variables. Anemia is quite common in older adults, with a prevalence of 11% in people aged 65+ years and many studies support this comorbidity as a significant risk factor for dementia [18,19], with an evident reflex on iron metabolism and other nutrients and, ultimately, a consequent increase in RDW [20]. Low cerebral hemoglobin associated with low oxygen levels can damage neurons and, consequently, lead to cognitive impairment. 

Concerning the number of lymphocytes, contradictory results have been reported in patients with Alzheimer’s disease (AD). The results did not indicate a reduced number of lymphocytes in patients who progressed to cognitive decline after one year of follow up, but an increase in functional decliners. No significant difference in the distribution of total lymphocytes between AD patients and healthy individuals was found in a previous study [21], though the decrease in the number of lymphocytes in AD is frequently reported [22,23]. This contradictory finding raises the need to further investigate the role of the lymphocyte in cognitive/functional decline. 

NLR and PLR are useful and low-cost biomarkers related to peripheral systemic inflammation [24] and integrate information from two leukocyte subtypes and platelets. In the present study, NLR was not elevated, but PLR was shown to be reduced in functional decline. It is known that an increase in the neutrophil count is often associated with the occurrence, progression, and severity of inflammation, while a decrease in lymphocyte count, as part of the regulatory immune barrier, is associated with the body’s response to stress [23]. Conversely, nutritional deficiencies, such as B12 deficiency, which is relatively common in older adults, can reduce the number of blood cells, including neutrophils [25]. A lower number of platelets was found only in individuals who presented functional decline after one year of follow-up. 

Most studies suggesting that peripheral leukocytes may be biomarkers for AD are opposed by others that show null effects or contrary results [26]. In this study, lower leukocyte count was associated with cognitive decline in SHAP, though with functional decline only in the univariate analysis. A possible explanation would be the loss of telomeric DNA from hematopoietic progenitor cells with aging. This finding implies that stem cell collections from an older patient may have compromised replicative capacity with a reduced response to hematopoietic growth factors [18]. 

After a year of follow up, the model showed that a reduced number of eosinophils may be associated with cognitive decline. On the other hand, in functional decline, a greater number of eosinophils and a decrease in the number of both basophils and monocytes were observed. Although the role of eosinophils in this context is not clear, it is known that basophils contain histamine, which has an anti-inflammatory character acting as a neurotransmitter in the central nervous system. However, the influence of AD on the number and function of basophils has remained elusive [19]. Concerning monocytes, it has been reported that this leukocyte plays a critical role in the pathogenesis of AD [27]. The model showed that in those who progressed to functional decline, monocytes were found to be less in number. 

Higher TG levels were the most important variable associated with cognitive decline in the model. Higher concentrations of plasma TG have been associated with an increased risk of non-AD dementia and ischemic stroke in other community-based epidemiological studies [28]. Bernath et al. [29] showed that long-chain, polyunsaturated fatty-acid-containing TGs (PUTGs) were significantly associated with MCI and AD. In addition, PUTG component scores were significantly associated with early-AD biomarkers, including hippocampal volume and entorhinal cortical thickness measured using MRI scans. Interestingly, although included in the final model, TG levels did not show relevance in the individual analysis of functional decline. 

Lower HDL-c levels were associated with both cognitive and functional decline. HDL-c and *APOE* have been associated with brain function, dementia, and AD in observational studies, mainly due to the apolipoproteins involved in the deposition and clearance of β-amyloid, a causal factor for neurodegeneration [30]. Larger studies indicate that plasma levels of HDL-c are inversely associated with the risk of dementia [30]. In our previous study, we also showed that lower HDL-c levels were independently associated with MCI and dementia when compared to controls in the Pietà study [31].

It was reported that brain insulin resistance or brain insulin reduction can accelerate AD pathologies such as neurofibrillary tangles and Aβ deposition [32], mainly mediated by insulin-like growth-factor (IGF) signaling, which can regulate the expression of tau protein and its phosphorylation [32]. Variable glucose levels did not show a linear relationship with cognitive or functional decline in the model, suggesting that its interaction with other peripheral markers could modulate the impairment in this older group. 

Many reports have shown that hypo-albuminemia (malnutrition) is associated with cognitive dysfunction [33,34]. It is also known that serum albumin is one of the most potent Aβ sequestering systems, which binds 90–95% of the Aβ in blood plasma [35]. However, lower albumin levels did not play an important role in cognitive decline since higher levels were observed in the model after one year of follow up. The hypothesis is that the increase in albumin levels could be related to a release toward the bloodstream in order to maintain the dynamic equilibrium of Aβ between the brain and blood plasma, as Aβ is increased in several dementia conditions [36]. 

Similarly, lower globulin levels were associated with cognitive and functional impairment in the model. Contrary to other results, increased globulin levels were shown to be associated with chronic subclinical inflammation commonly observed in dementia processes [37]. However, Dodel et al. [38] showed, in a clinical trial, that intravenous immunoglobulins might reduce the speed of metabolic decline in the medial temporal lobe in patients with AD, suggesting the beneficial effect of globulin on cognition and function in patients with dementia diseases.

GGT levels were reduced in both groups with cognitive or functional decline. Although related to the use of medications, low GGT levels do not represent important clinical variables. However, it is necessary to highlight that higher ALT levels related to cognitive impairment in the SHAP model and to functional decline in the univariate analysis as well as higher AST levels related to functional decline, were observed in the model. In fact, liver dysfunction is associated with the development of dementia, in addition to cardiovascular disease and insulin resistance. Through disruptions in glucose and lipid metabolism, key physiological functions of the liver also occur in the dementia process [39,40]. Nevertheless, Nho et al. [41] observed that increased AST to ALT ratio values and lower levels of ALT showed a significant association with reduced brain glucose metabolism, particularly in the orbitofrontal cortex and temporal lobes, areas of the brain implicated in memory and executive function. 

The creatinine levels showed a complex profile since higher levels were observed in cognitive decline and lower levels in functional impairment. Impaired kidney function may cause damaging effects on the brain by inducing cognitive impairment [42] and increasing the risk of dementia [43]. Impaired kidney function may cause vascular disease-related cerebral pathology, disturbances in water and electrolyte balance, vascular tone, oxidative stress response, and cytokine production, factors involved in cerebrovascular injury. However, in a follow up of 11.6 years from the Rotterdam Study including 69,790 people, impaired kidney function evaluated by estimated glomerular filtration rates (based on creatinine) was not related to a higher risk of dementia [44]. In addition, two baselines from the Atherosclerosis Risk in Communities (ARIC) Study, including 9967 participants aged 54 to 74 years, showed that lower estimated glomerular filtration rate (eGFR) based on cystatin C or β2-microglobulin, but not creatinine was associated with dementia [45]. Nevertheless, creatinine was noted to be a significant predictor of acute kidney injury in multiple studies that utilized ML models, showing that creatinine, a nonexpensive and accessible biomarker, could be applied to evaluate kidney function using algorithms [46,47,48].

Lower TSH levels are classically associated with dementia, corroborating the findings in the model. Cardiovascular risk factors may mediate the association between thyroid function and the development of dementia through vascular brain damage. Analyses were performed within the Rotterdam Study, comparing the TSH and free thyroxine levels with dementia [49]. Lower TSH levels were associated with higher dementia risk, independent of cardiovascular risk factors. Furthermore, the 10-year dementia risk decreased from 15% to 10% with higher TSH in older women and higher TSH was associated with better global-cognitive scores. Using random forest regression, Santhanam et al. (2022) observed that log TSH was a significant copredictor of fluid intelligence, a mental health parameter, in female young adults [50]. Another study showed that random forest performed the best to predict individuals with normal, high, and low levels of TSH, using ethnicity, free T4, anti-thyroid peroxidase (TPO) antibodies, free T3, body mass index (BMI), age, and anti-TPO as the most important feature in classifying TSH [51].

Our previous studies showed that the elevation in cortisol concentrations is associated with MCI and dementia, independent of *APOE* genotypes [31,52]. Differentially, the model did not show elevated cortisol levels in both cognitive and functional decline. Accordingly, Gil-Bea et al. also did not find higher concentrations of cortisol in individuals with MCI, mild AD dementia, or in the controls, suggesting that the increase in cortisol levels is not an initial event in dementia. Probably, elevated cortisol levels would be related to the progression of the established disease and would have no prognostic value, since the enhanced concentrations occur due to the loss of hypothalamus–pituitary–adrenal axis inhibition when there is already hippocampal damage [52,53]. Therefore, the results suggest that at the beginning of a cognitive and functional decline, cortisol levels are not elevated; however, they may increase as the disease progresses. The majority of studies, including ML, evaluated cortisol levels as an outcome, not a feature [54]. However, few studies included cortisol levels in ML algorithms able to predict different diseases, such as mental disorders [55], stress and dietary intake [56], and quality of life [57].

There is some expectation about the use of ML in laboratory medicine; however, there are some concerns that should be addressed, such as data quality, mainly missing data and label error; the cost of the computational infrastructure and the individuals with expertise to develop machine-learning algorithms, as well as ML standardization and regulation required for quality guarantee. At this time, best practices for the clinical validation of ML algorithms should be widely discussed [58,59]. 

It is important to recognize the limitations of the present study, such as the small sample size for each group and the cross-sectional measure of blood-based biomarkers. Although several drugs, hypertension, and diabetes mellitus frequencies were not different between the two groups, they may have interfered with the variables analyzed. Therefore, some apparent contradictory findings might be related to the fact that cognitive and functional impairment are heterogeneous entities since a clinical continuum from subjective decline to AD was observed in the dementia group. Although an external validation was not performed, an internal validation with accurate prediction was applied. 

## 5. Conclusions

The results suggest that routine laboratory tests present a good accuracy to predict cognitive and functional decline in an oldest-old population using ML models.

## Figures and Tables

**Table 1:** Demographic characteristics of the population and cognitive/functional performance in 2008–2009.

Characteristic	Population Evaluated (n = 132)
Age	79.52 ± 4.26
Male/Female	51/81
Cognitive decliners (n,%)	70, 53%
Functional decliners (n,%)	47, 36%
GSC score 2008	−1.73 ± 3.92
GSC score 2009	−2.15 ± 4.75
GSC score between 2008 and 2009	−0.41 ± 3.22
FAQ score 2008	1.57 ± 3.12
FAQ score 2009	3.23 ± 5.14
FAQ score between 2008 and 2009	1.66 ± 4.69

Functional Activities Questionnaire—FAQ, Global Cognitive Score—GCS. Values are expressed as mean ± standard deviation. Negative score: decliner; positive score: nondecliner.

## Data Availability

The data that support the findings of this study are available on request from the corresponding author, K.B.G.

## Supplementary Materials

The following supporting information can be downloaded at: https://www.mdpi.com/article/10.3390/brainsci13040690/s1, Table S1: Set of hyperparameters for each algorithm. Table S2: Routine laboratory variables measured at baseline in cognitive/functional decliners and non-decliners. Table S3: Performance of Machine Learning models applied in the study.
