# Supplementary material for: Machine Learning-Based Routine Laboratory Tests Predict One-Year Cognitive and Functional Decline in a Population Aged 75+ Years
Source: Brain Sci. 2023 Apr 20;13(4):690. doi: 10.3390/brainsci13040690 (PMC10137192; doi:10.3390/brainsci13040690)
Supplement: Supplementary file 1 [file brainsci-13-00690-s001.zip › brainsci-2261121-supplementary.pdf]

Supplementary Material

Table S1: Set of hyperparameters for each algorithm.

**Algorithm**	**Hyperparameters**
Random Forest	number of trees = {1, 2, 3, ..., 50}<br><br>depth of estimators = {1, 2, 3, ..., 20}
SVM	kernel = {rbf, linear}<br><br>C = {0.001, 0.01, 0.05, 0.1, 0.5, 1, 10}
XGBoost	number of trees = {1, 2, 3, ..., 50}<br><br>learning rate = {0.01, 0.05, 0.1};<br><br>max depth of estimators = {1, 2, 3, ..., 19}

SVM: Support Vector Machine

Table S2 – Routine laboratory variables measured at baseline in cognitive/functional decliners and non-decliners.

Variable	Cognitive decliners (n=70)	Non-cognitive decliners (n=62)	P value	Functional decliners (n=48)	Non-functional decliners (n=84)	P value
RBC / mm^3	4890285.71±464508.37	4944193.54±421712.15	0.488	4872195.12±477663.64	4924383.56±427089.58	0.549
Hemoglobin g/dL	14.14±1.18	14.50±1.19	0.079	14.22±1.27	14.25±1.14	0.874
Hematocrit %	43.56±3.34	44.51±3.52	0.111	43.88±3.50	43.89±3.40	0.990
Leukocytes /mm^3	5762.85±1447.85	5954.83±1282.75	0.424	5551.21±1466.99	6110.95±1409.15	**0.047***
Neutrophils %	57.34±8.50	58.50±9.40	0.461	55.68±7.92	58.69±8.14	0.057
Eosinophils %	3.45±3.70	3.83±3.56	0.548	3.56±3.36	3.393.63	0.812
Basophils %	0.62±0.56	0.67±0.47	0.594	0.58±0.63	0.73±0.44	0.170
Monocytes %	6.61±2.10	6.54±2.10	0.857	6.58±2.20	6.80±1.94	0.577
Lymphocytes %	31.88±7.58	30.27±8.28	0.245	33.46±7.64	30.31±7.57	**0.036***
Platelets /mm^3	221800.00±50808.99	219516.12±46816.60	0.789	208804.87±51607.27	237356.16±46282.75	**0.003***
RDW %	15.26±1.32	15.04±0.88	0.273	15.09±1.23	15.19±1.21	0.670
NLR	170.24±98.74	195.54±136.14	0.220	150.53±89.04	181.24±138.95	0.205
PLR	7463.28±2883.09	8300.21±5018.97	0.235	6678.25±2633.13	8528.11±3637.56	**0.005***
Fasting glucose mg/dL	112.32±31.49	114.54±30.09	0.680	113.17±38.78	116.32±36.88	0.667
Triglycerides mg/dL	139.37±57.07	118.90±68.77	0.064	125.58±55.72	142.21±71.17	0.199
HDL-c mg/dL	52.67±11.22	55.33±12.77	0.204	54.95±12.38	54.45±11.79	0.831

LDL-c mg/dL	139.41±39.30	133.70±28.13	0.335	134.85±37.02	142.84±30.95	0.220
TSH mU/L	2.81±4.34	2.38±2.01	0.295	2.92±3.08	2.10±1.75	0.132
AST U/L	23.64±11.88	22.37±5.95	0.447	25.90±15.24	22.02±4.79	0.132
ALT U/L	14.52±6.32	14.51±6.58	0.991	16.70±8.86	13.45±4.83	**0.034***
GGT U/L	35.98±25.58	37.85±32.77	0.713	37.09±29.32	36.80±30.91	0.960
Creatinine mg/dL	0.98±0.30	0.92±0.24	0.093	0.92±0.26	0.94±0.30	0.718
Albumin g/dL	4.18±0.20	4.13±0.27	0.220	4.15±0.30	4.16±0.21	0.799
Globulin g/dL	3.13±0.30	3.23±0.36	0.081	3.10±0.36	3.21±0.34	0.116
B12 vitamin pg/mL	492.78±442.02	409.25±248.79	0.177	472.07±372.20	415.27±276.55	0.356
Cortisol µg/dL	13.15±4.59	13.49±4.83	0.678	12.99±4.94	13.18±4.40	0.832

*RBC: red blood cell; RDW: red blood cell distribution width; NLR Neutrophil-lymphocyte ratio; PLR: platelet-lymphocyte ratio; AST: aspartate transaminase; ALT: alanine transaminase; GGT: gamma-glutamyl transferase; HDL-c: high-density lipoprotein cholesterol; LDL-c: low-density lipoprotein cholesterol; TSH: thyroid-stimulating hormone. Mean + standard deviation. *Significant: p<0.05.*

Table S3. Performance of Machine Learning models applied in the study.

Model	Sensitivity	Specificity	Accuracy
Cognitive decline			
Randon Forest	0.81	0.79	*0.79*
XGBoost	0.81	0.79	*0.79*
SVM	0.51	0.71	*0.71*
Functional decline			
Randon Forest	0.92	0.83	*0.92*
XGBoost	0.83	0.83	*0.83*
SVM	0.51	0.71	*0.71*

Sensitivity and specificity: weight average. SVM: Support vector machine.
